# Gluteal muscle metastasis with peritoneal dissemination from gastric cancer during postoperative adjuvant chemotherapy: a case report

**DOI:** 10.1186/s40792-021-01127-5

**Published:** 2021-02-06

**Authors:** Shotaro Korehisa, Akira Kabashima, Michihiro Ichimanda, Kenji Umeda, Hidenori Koso, Kazuhiro Yada, Motoki Arakane, Hideaki Anai

**Affiliations:** 1 Department of Surgery, National Hospital Organization, Oita Medical Center, 2-11-45 Yokota, Oita, 870-0263 Japan; 2 Department of Pathology, National Hospital Organization, Oita Medical Center, 2-11-45 Yokota, Oita, 870-0263 Japan

**Keywords:** Gastric cancer, Gluteal muscle metastasis, Peritoneal dissemination

## Abstract

**Background:**

Skeletal muscle metastasis from gastric cancer is rare and has a poor prognosis. We reported a case of gluteal muscle metastasis with peritoneal dissemination from gastric cancer during postoperative adjuvant chemotherapy.

**Case presentation:**

A 64-year-old man with gastric cancer underwent distal gastrectomy with D2 lymph node resection. The pathological diagnosis was poorly differentiated adenocarcinoma and signet cell carcinoma, T3N3bM0, Stage IIIC. Metastases were found in all regional lymph nodes, except 11p. The resection margin was negative. S-1 plus docetaxel therapy was administered as postoperative adjuvant chemotherapy. Six month post-operation, the patient presented with right gluteal muscle tenderness and abdominal distension. Computed tomography revealed a solid mass in the right gluteal muscle, a disseminated nodule on the abdominal wall, and massive ascites. Pathological examination of the gluteal muscle revealed signet cell carcinoma, similar to the resected gastric cancer. The tumor was diagnosed as gastric cancer metastases. Ascites cytology was class V. Thereafter, the patient underwent one course of capecitabine plus cisplatin combined with trastuzumab. Radiation therapy was also administered to relieve the pain of gluteal muscle metastasis. However, chemoradiotherapy was ineffective, and the patient died 2 months after the recurrence.

**Conclusions:**

Skeletal muscle metastasis and peritoneal dissemination during adjuvant chemotherapy indicated a poor prognosis.

## Background

Gastric cancer commonly metastasizes to the lymph nodes, peritoneum, and liver [1]. Skeletal muscle metastasis from gastric cancer is extremely rare, and its prognosis is poor [1]. The reason for the rarity of intramuscular metastasis remains unclear. High tissue pressure, accumulation of lactic acid, local changes in pH, and oxygenation were proposed causes for the rarity of this disease [2–4]. S-1 plus docetaxel adjuvant chemotherapy was reportedly effective in treating patients with stage III gastric cancer [5]. We reported a case of gluteal muscle metastasis with peritoneal dissemination from gastric cancer, discovered during S-1 plus docetaxel adjuvant chemotherapy.

## Case presentation

A 64-year-old man with gastric cancer underwent distal gastrectomy with D2 lymph node resection. Peritoneal dissemination or liver metastasis was not observed at the time of operation. The cancer invaded the anterior lobe of the transverse mesocolon. The resected specimen revealed a circumferential type 3 tumor at the antrum of the stomach.

**Fig. 1:**
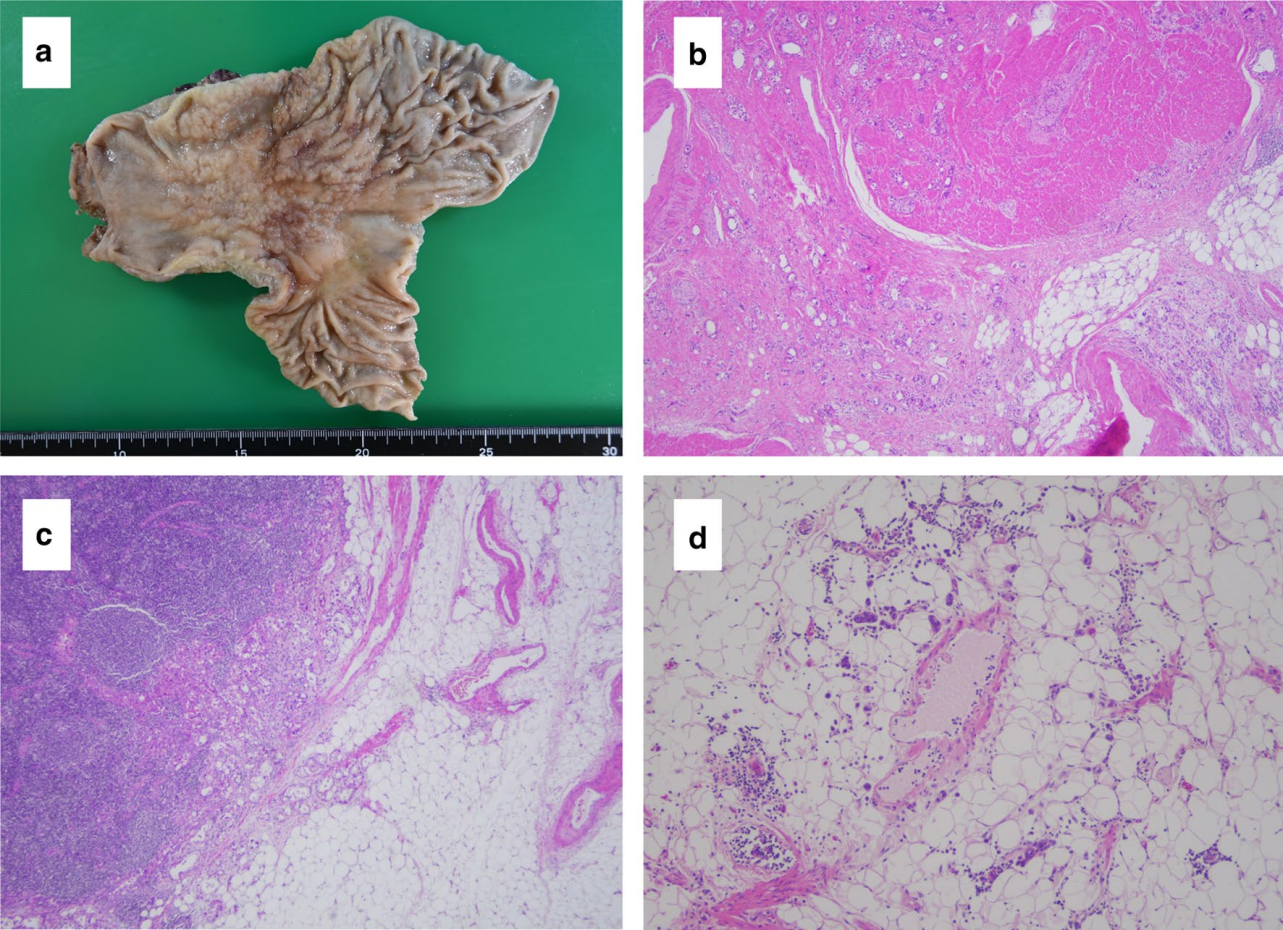
Macroscopic and microscopic appearance of the resected stomach. **a** Resected specimen reveals a circumferential type 3 tumor mainly at the antrum of the stomach. An ulcer scar can be seen in contact with the tumor. The oral and anal margins were 50 mm and 30 mm, respectively. The resection margin was negative. **b** Tumor invaded the subserosa, and there was no apparent exposure to the serosal surface (H&E staining, × 40) **c**, **d** Poorly differentiated adenocarcinoma invaded the adipose tissue from the lymphatic vessels and veins around the metastatic lymph nodes (H&E staining, × 100)

The oral and anal margins were 50 mm and 30 mm, respectively (Fig. 1a). Intraoperative ascites cytology was class II. The pathological diagnosis was poorly differentiated adenocarcinoma and signet cell carcinoma, pT3N3bM0, and pStage IIIC according to the Japanese Classification of Gastric Carcinoma 15th edition [6].

**Fig. 2:**
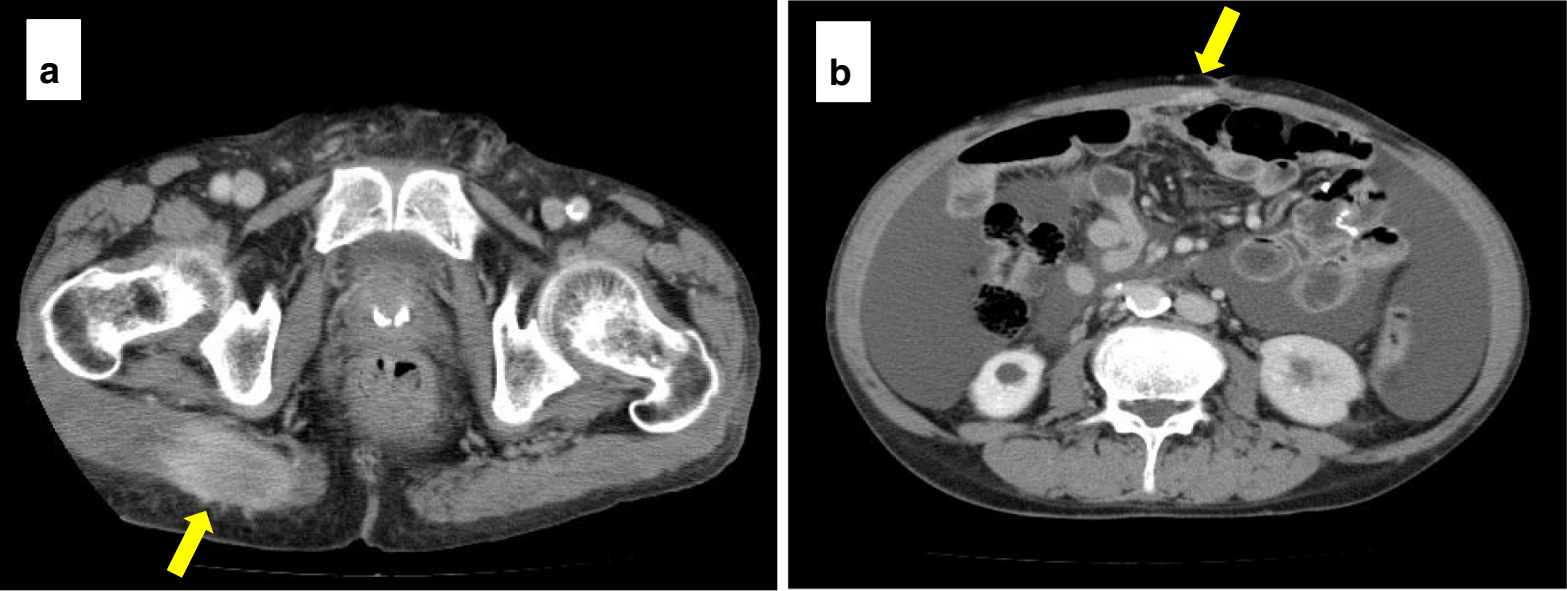
Abdominal contrast-enhanced computed tomography images 6 months after gastrectomy. **a** Computed tomography scan shows a solid mass (arrows) measuring 52 mm × 28 mm in the right gluteal muscle. **b** Computed tomography scan shows a disseminated nodule on the abdominal wall (arrows) and massive ascites

**Fig. 3:**
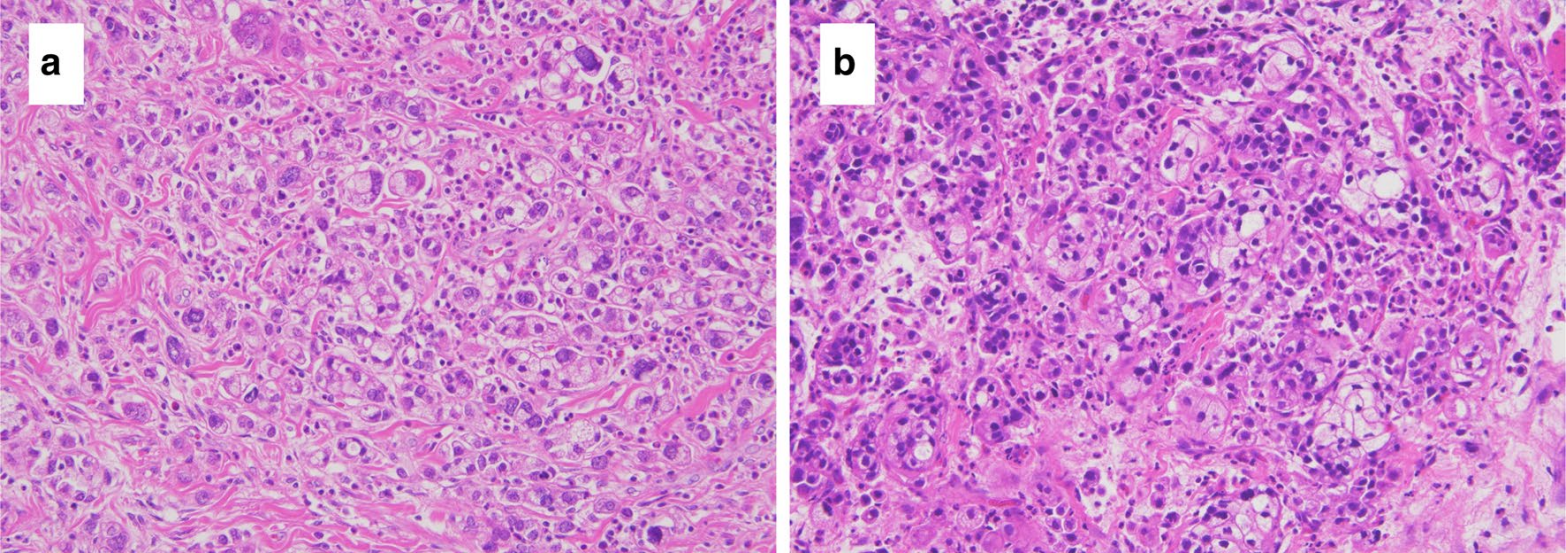
Microscopic appearance of the resected stomach and gluteal muscle mass. **a** Pathological specimens of the resected stomach reveals poorly differentiated adenocarcinoma and signet cell carcinoma (H&E staining, × 200). **b** Pathological specimens of the gluteal muscle mass reveals signet cell carcinoma, similar to the resected stomach (H&E staining, × 200)

**Fig. 4:**
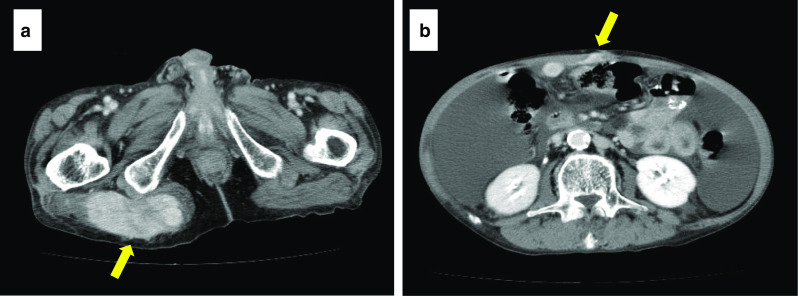
Abdominal contrast-enhanced computed tomography images 8 months after gastrectomy. **a** Enlarged right gluteal muscle metastasis (arrows). **b** Disseminated nodule on the abdominal wall (arrows) and increased ascites can be observed

The tumor invaded the subserosa, and there was no apparent exposure to the serosal surface (Fig. 1b). Lymphatic vessel and venous invasions were observed. Metastases were found in all regional lymph nodes, except 11p; they were found in 50 of the 58 harvested lymph nodes. Poorly differentiated adenocarcinoma invaded the adipose tissue from the lymphatic vessels and veins around the metastatic lymph nodes (Fig. 1c, d). The tumor had a strong tendency for invasion, and peritoneal dissemination was not observed. The resection margin was negative. S-1 plus docetaxel therapy was then administered as postoperative adjuvant chemotherapy. The patient was asymptomatic and in good general condition; S-1 plus docetaxel therapy was administered without any serious adverse events. Preoperative carcinoembryonic antigen (CEA) and carbohydrate antigen 19-9 (CA19-9) levels were 4.1 ng/ml and 49.2 U/ml, respectively. Three months after the operation, they were 9.2 ng/ml and 14.1 U/ml. Six months after the operation, they were 5.7 ng/ml and 13.0 U/ml. Postoperative CEA was slightly elevated. Six months after the operation, the patient presented with right gluteal muscle tenderness and abdominal distension. Computed tomography (CT) revealed a solid mass in the right gluteal muscle, a disseminated nodule on the abdominal wall, and massive ascites (Fig. 2a, b). No mass was noted in the skeletal muscles other than the right gluteal muscle. The patient could not undergo magnetic resonance imaging (MRI) and positron emission tomography (PET), because he was in deep pain. We performed an ultrasound-guided needle biopsy of the right gluteal muscle mass and an abdominal puncture. Pathological examination of the gluteal muscle biopsy revealed signet cell carcinoma, similar to the previously resected gastric cancer (Fig. 3a, b). Ascites cytology was class V. The right gluteal muscle mass was diagnosed as gastric cancer metastasis. Thereafter, the patient underwent one course of capecitabine plus cisplatin combined with trastuzumab, because the primary tumor was HER2-positive. Palliative radiotherapy was also performed to relieve the pain of gluteal muscle metastasis with a planned dose of 20 Gy. One week after the start of chemotherapy, the patient experienced impaired consciousness with rapidly progressing hyponatremia. Head computed tomography (CT) revealed no abnormalities. Two weeks later, he was diagnosed with the cisplatin-induced syndrome of inappropriate secretion of antidiuretic hormone (SIADH). Hypertonic saline was administered, and his impaired consciousness gradually improved. Radiotherapy was discontinued, and only one course of chemotherapy was administered due to adverse events. Chemoradiotherapy was ineffective, the right gluteal muscle metastasis enlarged, and peritoneal dissemination nodules progressively increased (Fig. 4a, b). The patient died 2 months after the recurrence.

Gastric cancer rarely metastasizes to the skeletal muscle, which is generally associated with widespread metastatic disease and poor prognosis [7–9]. Skeletal muscle metastasis from any primary tumor usually occurred multifocal and adjacent to the trunk (paravertebral muscles, gluteus, and thigh) and lower limb, followed by the arm, abdominal wall, and chest. Metastasis usually stem from lung (25%), genitourinary (21%), gastrointestinal (21%) and breast primaries (8.2%) [8–12]. Tuoheti et al. reported clinicopathological findings for 12 patients with skeletal muscle metastases of carcinomas such as lung, gastric, thyroid, uterus, and bladder. In two cases, the skeletal muscle metastases occurred as a solitary mass without any other clinically detectable metastases, whereas in the other 10 cases, the skeletal muscle metastases occurred as part of disseminated disease such as lymph node, visceral, and brain metastases [8]. In our case, gluteal muscle metastasis with peritoneal dissemination was observed 6 month post-operation. We reviewed the reported case of skeletal muscle metastasis from gastric cancer. We used the key words “gastric cancer” and “skeletal muscle metastasis” to search the literature. This revealed four cases of gluteal muscle metastasis from gastric cancer. Table 1 shows a review of gluteal muscle metastasis cases [1, 13–15].

**Table 1:** Review of reported cases of gluteal muscle metastasis from gastric cancer

Author	Year	Age	Sex	Interval (years)^a^	Other site of metastasis	Treatment after recurrence	Outcome
Kondo	2002	64	F	Synchronous	Peritoneal dissemination Adductor m	Excision Chemotherapy (5-FU)	Died (9 M)
Sakuma	2011	64	F	12	Peritoneal dissemination	Chemotherapy (SP)	Alive (18 M)
Pergolini	2014	67	M	Synchronous	Lumboaortic lymph node	Chemotherapy (FOLFIRI)	Died (3 M)
Aguirre	2019	57	F	10	Quadratus lumborum m Psoas m Vastus lateralis m Obturator internus m Piriformis m	Radiotherapy	Died (3 M)
Our case	2021	64	M	0.6	Peritoneal dissemination	Chemoradiotherapy (HXP)	Died (2 M)

M: male, F: female, m: muscle, 5-FU: 5-fulorouracil, SP: S-1 + cisplatin, FOLFIRI: 5FU + leucovorin + irinotecan, HXP: capecitabine plus cisplatin in combination with trastuzumab

^a^Duration between primary tumor resection and detection of metastasis

Two cases had synchronous metastases, while the remaining two cases had metachronous metastases. In the metachronous metastases cases, the durations between primary tumor resection and the detection of metastasis were 12 and 10 years, respectively. Our case was detected 6 months after gastrectomy. Pergolini et al. reported that skeletal muscle metastasis is often accompanied by synchronous metastasis to other organs [13].

Three patients had peritoneal dissemination. Two patients had multiple muscle metastases, and the remaining patients had solitary gluteal metastasis. One patient had lumboaortic lymph node metastasis. Our patient had solitary gluteal metastasis and peritoneal dissemination.

Pergolini et al. also reported that ultrasound-guided biopsy of the muscle was useful for diagnosis. In our case, the diagnosis of gluteal muscle metastasis was confirmed by ultrasound-guided muscle biopsy. In contrast, Kondo et al. reported that MRI was the most useful imaging modality for diagnosis [1]. However, our patient was restless and could not undergo MRI because of pain in the abdomen and buttocks. CT showed no muscle metastases other than the gluteal muscle.

Chemotherapy is the standard treatment for muscle metastasis of gastric cancer [16]. Radiotherapy is also effective in relieving pain and reducing the size of metastatic lesions [13]. Surgical excision reportedly relieved pain due to muscle metastasis [13]. In one of the cases, it was performed after chemotherapy to relieve gluteal pain [1]. In our case, surgical excision was not performed, because although it was a solitary lesion, the tumor was large, and the closing of the wound was expected to be difficult. Two patients, including ours, underwent palliative radiotherapy. Four patients, including ours, underwent chemotherapy. Therapy included 5-fluorouracil; a combination of S-1 and cisplatin; and 5-fluorouracil, leucovorin, and irinotecan. Recently, Yoshida et al. reported a phase III trial that demonstrated a significant clinical benefit of adding docetaxel to S-1 [5]. This combination was recommended as the standard postoperative adjuvant chemotherapy for patients with stage III gastric cancer. The most commonly reported sites for the first relapse after gastrectomy were the peritoneal sites, hematogenous sites, and lymph nodes. Significantly lower relapse rates were found in the S-1 plus docetaxel group than in the S-1 group for hematogenous sites (5.3% vs. 9.8%) and lymph nodes (4.8% vs. 11.3%). In contrast, no difference was found in terms of the incidence of local recurrence (0.4% vs. 0.4%) and peritoneal surface (9.3% vs. 12.9%). Aguirre et al. and Kamitani et al. reported two cases of metachronous skeletal muscle metastases, and these patients were administered postoperative adjuvant chemotherapy [15, 16]. In one case, adjuvant chemoradiotherapy with capecitabine was administered after total gastrectomy with D2 lymphadenectomy. The primary tumor was poorly differentiated from signet cell carcinoma of the esophagogastric junction, pT3N1M0, and pStageIIB. In another case, adjuvant chemotherapy with S-1 was administered after distal gastrectomy with D2 lymphadenectomy. The primary tumor was poorly differentiated carcinoma of the pyloric antrum, pT2N1M0, and pStageIIA. Left latissimus dorsi muscle metastasis occurred 12 months after the operation. In our case, skeletal muscle metastasis with peritoneal dissemination was observed during S-1 plus docetaxel therapy. This is the only case of early skeletal muscle metastasis during postoperative chemotherapy. After recurrence, our patient underwent one course of capecitabine plus cisplatin combined with trastuzumab.

If there was peritoneal dissemination and the stage was IV at the time of operation, capecitabine plus cisplatin combined with trastuzumab therapy might have been administered after the operation.

Studies suggested that S-1-containing chemotherapy was ineffective in patients who showed S-1 adjuvant failure [17, 18]. Conversely, several clinical trials have documented the efficacy of molecularly-targeted drugs, such as trastuzumab and ramucirumab [19, 20]. Recurrence occurred while using S-1 plus docetaxel therapy. Ramucirumab plus paclitaxel combination therapy was not performed in our case. However, chemoradiotherapy was ineffective, and the patient died 2 months after the recurrence. Among the reported cases, three patients died, and the median survival time was 3 months (range, 3–9 months). One patient remained alive for 18 months after recurrence. A combination of S-1 and cisplatin achieved a complete clinical response in this case. We expect to develop an effective postoperative adjuvant chemotherapy for peritoneal dissemination and rare skeletal muscle metastases.

## Conclusion

We reported a rare case of gluteal muscle metastasis with peritoneal dissemination during adjuvant chemotherapy. The prognosis of patients with skeletal muscle metastases remains poor.

## Data Availability

The data sets supporting the conclusions of this article are included in the article.

## Abbreviations

CEA: Carcinoembryonic antigen
CA19-9: Carbohydrate antigen 19-9
CT: Computed tomography
MRI: Magnetic resonance imaging
PET: Positron emission tomography
SIADH: Drug-induced syndrome of inappropriate secretion of antidiuretic hormone
